# Factors influencing autumn–winter movements of midcontinent Mallards and consequences for harvest and habitat management

**DOI:** 10.1002/ece3.10605

**Published:** 2023-10-25

**Authors:** Aaron T. Pearse, Michael L. Szymanski, Cynthia A. Anchor, Michael J. Anteau, Rocco M. Murano, David A. Brandt, Joshua D. Stafford

**Affiliations:** ^1^ U.S. Geological Survey, Northern Prairie Wildlife Research Center Jamestown North Dakota USA; ^2^ North Dakota Game and Fish Department Bismarck North Dakota USA; ^3^ Department of Natural Resources Management South Dakota State University Brookings South Dakota USA; ^4^ South Dakota Game, Fish and Parks Brookings South Dakota USA; ^5^ U.S. Geological Survey, South Dakota Cooperative Fish and Wildlife Research Unit Brookings South Dakota USA

**Keywords:** autumn, distribution, harvest, Mallard, migration, movement, winter

## Abstract

Annual phenology and distributions of migratory wildlife have been noticeably influenced by climate change, leading to concerns about sustainable populations. Recent studies exploring conditions influencing autumn migration departure have provided conflicting insights regarding factors influencing the movements of Mallards (*Anas platyrhynchos*), a popular game species. We determined factors affecting timing and magnitude of long‐distance movements of 97 juvenile Mallards during autumn‐winter across the midcontinent of North America marked with implanted transmitters in North and South Dakota, 2018–2019. Factors influencing variation in movement timing, along with direction and magnitudes, depended on type of movement (i.e., regional [25–310 km], initial migration, or subsequent migration movements [>310 km]). Photoperiod influenced probability of initiating all movements, although the effect was most influential for regional movements. Minimum temperature most influenced initial migration events (probability of movement increased 29% for each 1°C decrease); favorable winds also increased likelihood of initial migration events. Probability of subsequent migration events increased 80% for each 1 cm increase in depth of snow. Subsequent migration movements also were 2.0 times more likely to occur on weekend days, indicating disturbance from humans may influence movements. Migration distances increased 166 km for each 1°C reduction in minimum temperature. We also observed markedly different autumn‐winter distributions of marked birds between years. Median locations during autumn‐winter 2018–2019 were ~250 km farther north and ~300 km farther west during mid‐December–January compared to the same time in 2019–2020. Concurrently, harvest rates for marked females and males were 10% and 26% during autumn‐winter 2018–2019 and 26% and 31% during autumn‐winter 2019–2020. Climate‐related changes may result in increasingly variable autumn‐winter distributions, with implications for wildlife recreationalists, conservation planners, and harvest managers.

## Graphical abstract

We determined factors affecting timing and magnitude of long‐distance movements of juvenile Mallards during autumn‐winter across the midcontinent of North America marked with implanted transmitters in North and South Dakota, 2018–2019. We found a combination of exogenous (temperature, wind, snow cover, disturbance) and endogenous (photoperiod) factors influenced variation in movement decisions, with direction and magnitudes depending on type of movement.

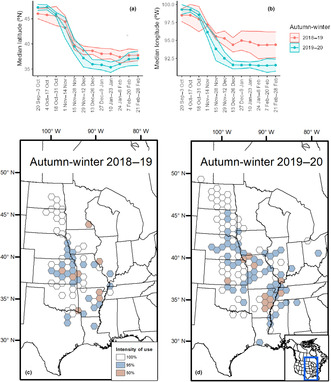

## 1 INTRODUCTION

Movements are a key facet of avian ecology that directly influence ecological patterns such as population distribution and density (Nathan et al., 2008). Movements occur between locations at distances relative to a hierarchy of habitat selection (Johnson, 1980), where birds might move moderate distances within a settled region (e.g., third‐order), or longer distances between regions indictive of migratory decisions to settle new regions (e.g., second‐order). All movements, regional or migratory, can be shaped by genetics, physiology, behavior, and ecology of populations and species (Newton, 2007). In the Northern Hemisphere, onset of autumn migration marks the beginning of a nonbreeding period in which individuals may undergo events of growth and maintenance, rejuvenation (e.g., feather molt), nuptial behaviors, and, ultimately, preparation for spring migration. Birds accomplish these annual events by seeking locations with resources that promote a favorable energy balance, allowing them to forage and avoid disturbance, disease, and predation.

Many hypotheses have attempted to explain avian movement and migration decisions that allow individual birds to exploit seasonally available resources. Changes in abundance and availability of critical resources have been considered primary factors influencing migration phenology (Newton, 2007). The concept of resource tracking generally identifies food as one important resource that may influence movements, wherein animals attempt to match their location to current and anticipated resource availability (Abrahms et al., 2021). Resource availability for waterbirds becomes greatly reduced or eliminated when surface waters freeze, prompting movements out of the area (Jorde et al., 1984; Nuijten et al., 2013). Energy conservation serves as a strong motivation for choosing areas that balance thermoregulation demands at stopover sites with costs of foraging and flight (Wikelski et al., 2003). Environmental conditions that energetically favor long‐distance movements (e.g., tailwinds) have been identified as influencing migration timing (Burnside et al., 2021; Kölzsch et al., 2016; O'Neal et al., 2018), and waterbirds may make local movements in response to perceived or real risk to survival (Madsen & Fox, 1995).

The Mallard (*Anas platyrhynchos*) is an abundant and widespread duck species that exhibits considerable variation in migratory behavior due to a relatively large body size and behavioral plasticity in habitat use. Across North America, many Mallard populations have been identified as facultative partial migrants, indicating migration behavior and distance moved relate to environmental conditions and available resources (Drilling et al., 2020; Newton, 2007). In North America, Mallards typically have a protracted autumn migration and are identified as one of the last species of ducks to leave northern breeding latitudes (Krementz et al., 2012). In certain years and locations, some individuals remain at higher latitudes during the nonbreeding period, enduring extreme weather conditions if adequate resources remain accessible (Olsen et al., 2011; Spivey et al., 2017). Migration timing and local abundance have been associated with combined conditions of below‐freezing ambient temperatures and snow cover, conditions which have been hypothesized to induce movements by affecting cost of thermoregulation and food availability (Schummer et al., 2010; Weller et al., 2022).

Wildlife professionals and their constituents are keenly interested in understanding what drives autumn‐winter movements, migration events, and distribution of midcontinent Mallards (Kaminski et al., 2005; Schummer et al., 2019). Understanding factors influencing migration and movements of Mallards has societal interest because Mallards are a preferred species of waterfowl by hunters and have the largest annual harvest of all duck species in the United States (Patton, 2018; Raftovich et al., 2019; Schummer et al., 2019). More generally, waterfowl movements and migrations have shaped aspects of past and contemporary human cultures and traditions, connecting humans and wildlife by affecting societal uses, desires, and values of wildlife (Duebbert, 2003; Hochbaum, 1955). Well‐known species, such as the Mallard, have also stimulated broader interests in contemporary issues, such as biodiversity and the value of citizen science (e.g., Wiegers et al., 2022). The societal value of the Mallard is great enough that the 2012 North American Waterfowl Management Plan explicitly linked people, waterfowl populations, and habitat (Humburg et al., 2017), acknowledging that participation in waterfowl hunting and desire for abundant waterfowl populations influences funding for waterfowl habitat conservation.

Climate‐induced changes in winter distribution of ducks can affect conservation delivery and hunting in nonbreeding areas (Meehan et al., 2021). Distributions of migratory ducks are a direct result of the decisions, distances, and directions of the types of movements that individual birds make (i.e., migration and regional movements). Data describing distribution of individual live Mallards in time and space are uncommon, and other descriptions of distributions can be limited by survey coverage or include only locations where birds are harvested. Recent studies exploring conditions influencing migration departure decisions provide conflicting insights as to which factors influence movements of Mallards during the autumn‐winter period when hunting occurs. O'Neal et al. (2018) suggested that ducks timed migration events to weather conditions favorable to flight (i.e., tailwinds, no precipitation, clear skies), whereas Weller et al. (2022) suggested that adult Mallards migrated in response to food availability and thermoregulation. In an effort to reconcile this discrepancy and expand our understanding beyond migration departure decisions, we quantified factors influencing probability of departure and distance of regional and migration relocations during autumn‐winter by juvenile Mallards originating from breeding areas in North and South Dakota. Our study of juvenile birds provides novel information on how birds make movement decisions during their first nonbreeding period when they rely on a combination of innate behaviors (e.g., migratory restlessness) and conspecific cues (e.g., departure of other birds in their social group). Moreover, information regarding how Mallards concurrently make movement decisions and distribute themselves would be useful for effective habitat management and to elucidate potential consequences of climate change that may influence hunter success across multiple latitudes (Notaro et al., 2016). We also considered consequences of distributions of juvenile Mallards arising from their movement decisions by reporting harvest rates of birds marked during our study between two autumn‐winter seasons across the midcontinent of North America.

## 2 METHODS

### 2.1 Study area

**FIGURE 1:**
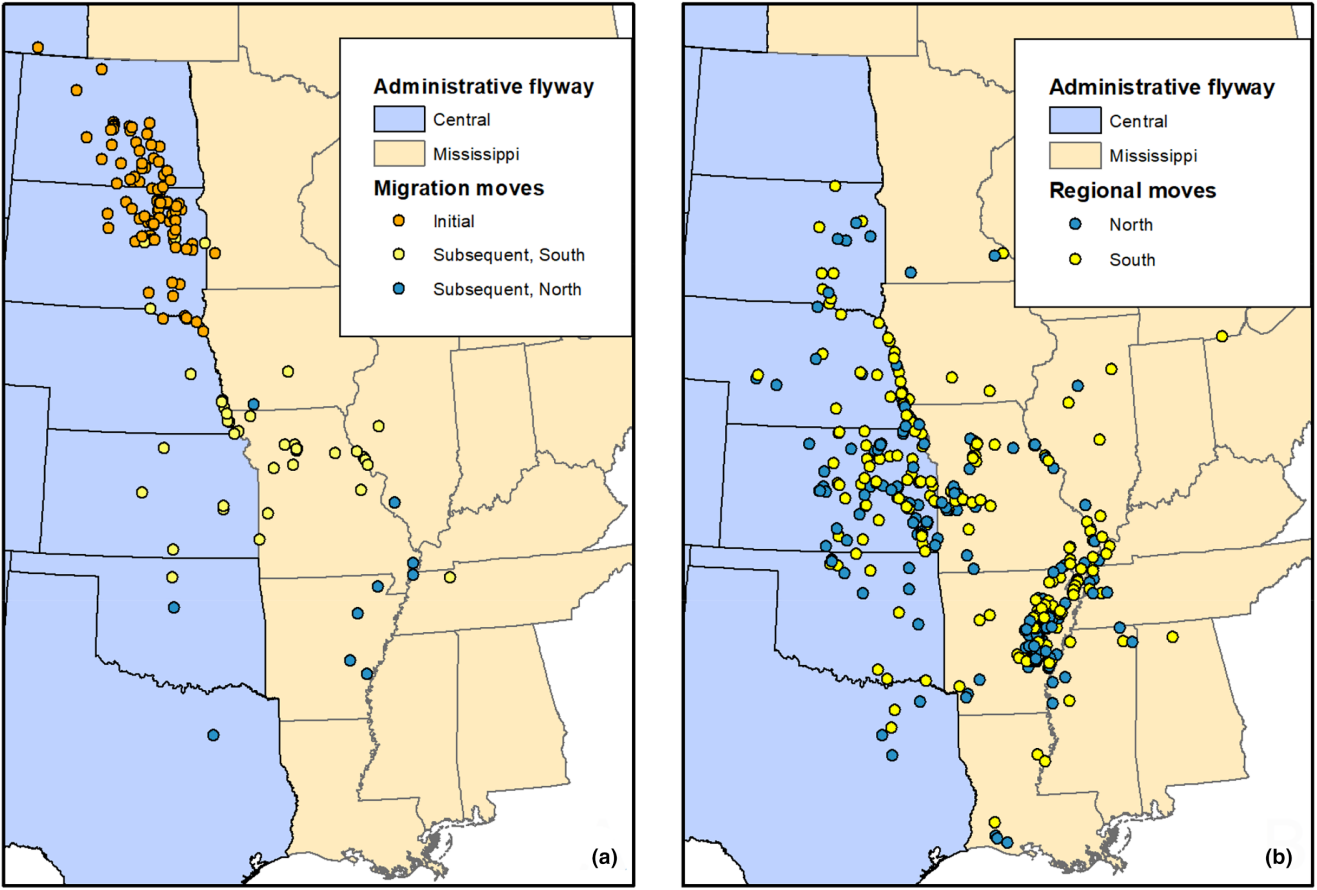
Origins of initial and subsequent migrations (a) and regional (b) movements made by juvenile Mallards captured at sites in North and South Dakota, USA, 2018–2019.

We captured juvenile Mallards across 14 counties in the Prairie Pothole Region of North and South Dakota during 2018 and 2019 (Figure 1). Capture locations were in a historically mixed‐ and tall‐grass prairie landscape with high densities of glacially formed wetlands (Doherty et al., 2018; Stewart & Kantrud, 1971). This region included generally flat lands with gently rolling topography in the glaciated plains, and lands of more uneven terrain with hummocks in the Missouri and Prairie Coteau regions. Land use was dominated by crop production and livestock grazing (Doherty et al., 2013; Samson & Knopf, 1994; Wright & Wimberly, 2013). We tracked marked birds throughout a 2.1‐million km^2^ area in the midcontinent region of North America (i.e., the Central and Mississippi administrative flyways; Figure 1). Mallards used autumn and winter locations in 19 U.S. States (Alabama, Arkansas, Illinois, Iowa, Kansas, Kentucky, Louisiana, Michigan, Minnesota, Mississippi, Missouri, Nebraska, North Dakota, Ohio, Oklahoma, South Dakota, Tennessee, Texas, Wisconsin) and the Canadian Province of Saskatchewan (Figure 1).

### 2.2 Location data

We captured flightless and recently fledged juvenile female and male Mallards during 2–30 August 2018, and 16 July–30 August 2019 using nightlighting, drive trapping, baited swim‐in traps, and rocket‐netting. Our work focused on juvenile Mallards because almost no research has been conducted on hatch‐year ducks during the postfledge period, yet they differ from adults in important ways. During this period juvenile Mallards experience unique physiological changes, learn to fly, are more vulnerable than adults to predators and harvest, and likely require diverse resources to meet demands of feather growth, energetic requirements, and accumulation of lipid stores (Alisauskas et al., 2014). We marked 56 flightless birds (28 in 2018 and 28 in 2019; ≤2 per capture location) of estimated ages between 5.0 and 6.5 weeks old and body masses of 640–1080 g. We also captured 81 birds (29 in 2018 and 52 in 2019; ≤5 birds per location) that were newly flighted or capable of limited sustained flight, based on primary feathers that were still growing; body masses of recently flighted Mallards were 741–1330 g. Recently flighted birds were marked at 8 preseason banding sites in 2018 and 15 sites in 2019. Transmitters weighed 32 g and our target mass of telemetered Mallards was ≥640 g to ensure transmitters were ≤5% of body mass at marking. We also considered that birds would increase in size and mass during their first autumn‐winter (e.g., to 1000–1100 g or ≤3.2% of body mass; Drilling et al., 2020; Szymanski et al., 2013), further reducing any potential negative consequences of marking (Caccamise & Hedin, 1985). We marked all birds with a standard size 7A U.S. Geological Survey (USGS) band on one leg and an auxiliary band on the other leg that included contact information for the North Dakota Game and Fish Department or South Dakota Department of Game, Fish and Parks, depending on the location of capture.

Captured birds were immediately transported to a nearby (≤1.5 h travel time) mobile surgical facility for intracoelomic implantation of a 32 g GPS/GSM transmitter (OrniTrack‐130 3G; Ornitela) with a percutaneous antenna (Mulcahy & Esler, 1999; Petersen et al., 1995). All surgeries were performed by licensed wildlife veterinarians, who had experience and training in the implantation procedure. Juvenile Mallards were anesthetized with vaporized isoflurane, administered bupivacaine (2 mg/kg, local anesthetic) and butorphanol (1 mg/kg; spectrum analgesic), and intubated during surgeries. Marked birds were administered 20 mL of Lactated Ringer's solution subcutaneously postsurgery and held for recovery ≥1 h before transportation to capture locations for release. Capture and marking protocols were approved by South Dakota State University's Institutional Animal Care and Use Committee (Approval #18‐048A) and all activities were conducted under USGS Bird Banding Lab permits 06824 and 06897 in addition to appropriate state and federal permits.

We programmed transmitters to collect between five and eight GPS locations per day while birds were north of 43° N latitude as part of an assessment of postbreeding habitat use, and locations every 1 or 2 days in areas south of 43° N. Because transmitters provided location data at varying temporal resolutions during autumn‐winter, we standardized relocations to one location per day by randomly selecting one location to represent each day under observation when more than one location was available. We censored data collected within 14 days of transmitter deployment for all birds and within 10 days of the last bait dispersal at a site to minimize capture, surgical, or baiting effects. We also limited our analyses to data collected between 20 September and 28 February each autumn‐winter (2018–2019 and 2019–2020), reflecting the temporal range of hunting seasons and the autumn‐winter migratory period. We removed locations from analyses if internal body temperatures were <39 or >46°C, which were indicative of mortality. We also removed locations with an instantaneous velocity of >1.4 m/s, as these locations were likely when birds were in flight (Byrne et al., 2017). Accuracy assessments of similar transmitter types infer an average horizonal accuracy of <8 m (Byrne et al., 2017).

We used a k‐means cluster analysis to identify groupings in daily movement distances that minimized variance within groups (Dubes & Jain, 1988). We square‐root transformed daily movement distance before conducting analyses, because k‐means procedures can be sensitive to positively skewed data (Moorter et al., 2010; Steinley, 2006). We identified the fewest number of clusters that best described the distribution of daily movement distances using the elbow method to visually inspect how the total within cluster sums of squares decreased by identifying two to eight clusters and choosing the number of clusters at the inflection point (Pham et al., 2005). We found that three clusters provided an adequate categorization of movement distances, and we interpreted the increasing magnitude of movement distances as representing generally local, regional relocation, and migration movements (Figure S1). We used the minimum and maximum values for each categorized cluster to identify threshold distances that separated clusters of movement distances. We used these threshold distances to identify groupings of days where birds were at local‐use areas, with longer regional or migration movements separating areas of local use (Figure S1). We calculated a geographic centroid for each local‐use area and determined timing of arrival and departure and days present at local‐use areas. Using Euclidean geometry, we calculated the distance and movement bearing between local‐use areas. Finally, we identified and categorized initial migration moves from all subsequent migration moves for each bird. For movement analyses, we included all migration movements and shorter regional movements occurring after the initial migration move for each marked bird. We categorized regional movements as occurring early or late in the autumn‐winter period by using the median timing of regional moves occurring after the initial migration move for each bird. We performed analyses in program R version 3.6.0 (R Core Team, 2019).

### 2.3 Movement timing

We used a time‐to‐event framework to investigate factors influencing variation in timing of regional and migration movements during autumn‐winter (Oppel et al., 2009; Xu & Si, 2019). We could not observe exact timing of all movement events because of missing daily location data, which created uncertainty in the exact timing of movements. Therefore, we used a logistic‐exposure model that relaxed assumptions of requiring equal exposure periods (1 day) or knowing the precise timing within an exposure period when an event occurred (Shaffer, 2004).

#### 2.3.1 Predictor variables

Many hypotheses have been identified to explain timing of migration and other large movements. We used these hypotheses to develop models that might be useful for understanding autumn‐winter movement decisions of Mallards in the midcontinent region of North America (Table S1). Various aspects of migration have been found to be under endogenous control, allowing naïve birds to gain physiological preparedness and migrate to novel locations and environments. Photoperiod may trigger migratory restlessness in birds, along with controlling other physiological and behavioral phenomena related to migration (Newton, 2007; Van Den Elsen, 2016). Therefore, we included day‐ and location‐specific photoperiod as a factor potentially influencing timing of movements and hypothesized an inverse relationship between photoperiod and movement decisions. We derived photoperiod based on spatial coordinates and day of year as implemented in the R package geosphere using the “daylength” function (Hijmans et al., 2019).

Environmental conditions have strong influences on timing of migration and other movements of birds (Newton, 2007). All environmental information was acquired from the Movebank automated system (Dodge et al., 2013) for each specific location (0.3° block spatial resolution) and day at 3‐h time intervals, which originally were derived from National Centers for Environmental Prediction North American Regional Reanalysis (NCEP‐NARR) data. Ambient temperature can serve as information to nonbreeding birds regarding decisions of when and why they may leave a specific area, which can correspondingly influence winter distributions of Mallards (Nichols et al., 1983). Thermoregulatory costs can be high when ambient temperatures are outside of the thermoneutral zone, potentially causing birds to move to locations with more favorable conditions (White et al., 2007). Moreover, wetland‐dependent species that require open water may use changes in ambient temperatures as a signal of eventual loss of this critical resource once temperatures are sustained below freezing (Xu & Si, 2019). We derived minimum daily temperature (°C) for each used location.

Birds have an exceptional ability to move in response to seasonally abundant or scarce resources (Abrahms et al., 2021). For example, Mallards may leave locations when available food is covered by snow, rendering unavailable residual grain they are known to exploit (Baldassarre & Bolen, 1984; Bossenmaier & Marshall, 1958). We hypothesized that departure probability of marked Mallards would increase when snow buried food resources and used NCEP‐NARR data to derive daily snow depth at used locations (daily maximum value, cm).

Migratory birds may time movements during the absence of poor flight conditions caused by precipitation and cloud cover (O'Neal et al., 2018; Schaub et al., 2004) or during favorable wind velocity and direction (Åkesson et al., 2002). We used NCEP‐NARR daily values of total precipitation accumulated at surface (cm) to designate potential poor conditions that might decrease the likelihood of a regional or migration movement. In addition, we calculated average percentage cloud cover throughout each day based on the 3‐h maximum of high‐ (>20,000 ft above ground level [AGL]), mid‐ (6500–20,000 ft AGL), and low‐level (<6500 ft AGL) cloud layers, because we hypothesized departure timing might be delayed by presence of cloud cover (Beason, 1978; O'Neal et al., 2018). We also hypothesized that tailwinds of increasing speed would be associated with greater rates of movement of marked individuals. To investigate effects of wind on movement timing decisions, we calculated bearing and magnitude of daily wind vectors from the mid‐day daily velocity (m/s) of the north–south (meridional) and east–west (zonal) components of wind at 10 m above the surface from NCEP‐NARR data. We then added this daily wind vector with the regional or migration movement vector, where the direction was the movement bearing (degrees) and the magnitude was the maximum daily wind speed across all observations. The magnitude of the resultant vector, relative to the maximum wind speed, provided an index of wind‐aided movement. For example, a value of −1.0 represented a daily wind vector in an exact opposing bearing, and 1.0 represented a daily wind vector exactly equal to the ultimate regional or migration movement bearing if wind velocity equaled the maximum of daily values.

Human activity influences bird distribution and behavior and hunting specifically has been found to be an activity that can have considerable influence on waterfowl movements and local abundance (Dooley et al., 2010; Madsen & Fox, 1995; Madsen, 1998). To investigate an association between disturbance from hunting activity and departure decisions, we would ideally use spatiotemporally explicit information on hunting activity. Unfortunately, these data are not collected in a consistent manner that spans multiple states, except for hunter diary surveys conducted by the U.S. Fish and Wildlife Service (Raftovich et al., 2019). However, this survey does not sample enough hunters across space (county‐level) or time (daily) to elicit information that would be useful for our purposes. Lacking an explicit metric for spatiotemporal variation in hunting disturbance across the midcontinent of North America, we used the designation of each day as occurring on a weekend (i.e., Saturday or Sunday) or weekday (i.e., Monday–Friday) to compare the relative probability of a bird leaving their immediate area to initiate migration or make a regional or subsequent migration movement. We justified this proxy of relatively different disturbance on weekends compared to weekdays based on multiple factors. First, Americans spend more time on leisure activities on weekends compared to weekdays (U.S. Bureau of Labor Statistics, 2023), and studies have found increased hunting effort occurring on weekends (Evans & Day, 2002; Gleason & Jenks, 1997; MacDonald et al., 1999; Mikula et al., 1972; Reynolds & Bishop, 1979). During August Management Take and Early September Canada goose Seasons in North Dakota in 2018 and 2019, 51% of combined reported hunting days occurred on weekends (Szymanski et al., 2019; Szymanski & Stechmann, 2020). We observed 51% (19 of 37) of harvested telemetered ducks recovered on weekends (1.8 times more likely than weekdays); the probability of this result occurring by random chance alone was 0.2% or 1 in 525. These limited results are further supported by a sample of juvenile Mallards banded and recovered in 2018 and 2019 in North and South Dakota; 49% of harvested birds were recovered on weekends (1.7 times more likely than on weekdays), supporting the notion that more hunting occurred on weekends compared to weekdays throughout autumn and winter and across a large portion of the Central and Mississippi Flyways in the United States. Our use of this proxy does not assume that all harvest or disturbance occurred on the weekend, only that there is evidence to indicate that waterfowl hunters were relatively more abundant and active on the weekends than weekdays. Moreover, greater outdoor recreational activity of all types occurs on weekend days compared with other days of the week (Vaara & Matero, 2011), further supporting our use of weekends as a potential index of disturbance. Because timing of movements was uncertain in some instances, we calculated this predictor variable as the proportion of days as Saturday or Sunday, where if the interval was a single day, the predictor simplified to 0 or 1. Finally, we did not consider holidays as weekend days, because of the variability in work schedules on holidays; thus, this decision constituted a conservative approach to quantifying potential temporal variation in human disturbance.

#### 2.3.2 Data analyses

We conducted separate modeling efforts for regional and migration movements. We fit models using the logistic‐exposure procedure as implemented in program R version 3.6.0 (R Core Team, 2019) with the glmer function to fit models and estimate parameter effect sizes (Bates et al., 2015; Shaffer, 2004). Model coefficients were initially fit and reported as the probability of birds remaining at a location; we report predictions and interpret results as probability of departure. In all models, we included sex as a fixed effect and bird identification as a random effect to account for multiple movements for each bird. We inspected correlations among spatial and temporal covariates by calculating correlation coefficients. If relationships between covariates were strong (|*r*| > .6), we restricted use of both variables in the same model (Table S2). We developed 10 models based on hypotheses. Models described hypotheses relating to photoperiod, ambient temperature, snow cover, ambient conditions precluding movement (precipitation and cloud cover), ambient conditions promoting movement (favorable wind), and disturbance indexed by weekend days. We also included more complex models where we included all predictor variables to test if multiple rather than single hypotheses were related to movement timing. We included one model with all covariates as additive factors and interacting with time, latitude, or direction to test whether the influence of predictor variables varied. For migration movements, we categorized time by identifying migration movements occurring initial or subsequent by individual birds. For regional movements, we categorized movements based on the median date of all regional movements used in analyses (i.e., those occurring after the initial migration move for each bird) or a categorical direction metric identifying either a northerly or southerly regional movement. We used Akaike's Information Criterion (AIC) values to rank models and considered those with ΔAIC < 2 as competitive (Burnham & Anderson, 2003). For competing models, we investigated individual predictor variable effects by inspecting the magnitude and 95% confidence interval of parameter estimates. We also calculated a relative likelihood (RL) of model parameters by fitting nested models where we sequentially removed each variable from the model and calculated RL = exp([AIC_reduced model_ − AIC_full model_]/2) and interpreted RL as the strength of evidence of the predictor variable on the response (Galipaud et al., 2017). We scaled continuous predictor variables by subtracting the mean and dividing by the standard deviation (Table S1).

To illustrate model results, we back‐transformed logit estimates into daily probabilities of movement (1–Probability of remaining) and calculated 95% confidence intervals of predictions. We held other values at their means when predicting relationships as a function of select predictor variables unless noted otherwise.

### 2.4 Movement distance and direction

For each set of the migration and regional movements used in the aforementioned analyses, we used the same multimodel inference procedure to test hypotheses that may influence distance moved. We fit models using general linear mixed models as implemented in program R version 3.6.0 (R Core Team, 2019) with the lmer function to fit models and estimate parameter effect sizes (Bates et al., 2015). We included the same 10 models as used in the timing analyses. We inspected individual predictor variable effects of competing models by inspecting the magnitude and 95% confidence interval of slope parameter estimates and RL to gauge relative importance. For migration moves, we calculated the difference in the minimum and maximum daily temperatures birds experienced on the day departing sites from the first day at arrival sites. We also correlated these temperature differences with distance of migration movements. We summarized bearings for migration and regional movements, plotted them in circular plots with distances of movements, and categorized them into four cardinal directions (i.e., northeast, northwest, southeast, southwest).

### 2.5 Annual distribution and harvest rates

We calculated median latitude and longitude of daily locations for each bird during each 2‐week period (fortnight) between 20 September and 28 February by autumn‐winter. To depict large‐scale changes in distribution each year, we plotted median latitude and longitude by fortnight and autumn‐winter. In addition, we used a hexagonal grid (50‐km side length) to sum all locations (weighted by the number of days each location represented), including day of first migration until transmitter failure, death, or end of the autumn‐winter period within each grid cell. We summarized grid cells containing 50%, 95%, and 100% of bird‐use days each autumn‐winter to depict annual differences in intensity of use.

We identified death of marked birds if internal temperature sensor readings dropped below typical body temperature and if movement ceased. We also used harvest information reported to the USGS Bird Banding Laboratory from the standard USGS band or to the North Dakota Game and Fish Department or South Dakota Department of Game, Fish and Parks as indicated by the auxiliary leg band. Using the two leg bands, we assumed high reporting rate that did not vary between hunting seasons (Arnold et al., 2020). We summarized the fate of all individuals during their first hunting season and calculated simple metrics of direct harvest occurring in North and South Dakota (i.e., natal areas) and other states where birds were reported to be harvested.

## 3 RESULTS

We captured and implanted transmitters in 137 Mallards during July–August 2018 and 2019. Of the 123 birds that provided usable movement data, our sample included 55 females and 68 males, 47 flightless and 76 flighted juvenile birds, 73 birds captured in North Dakota, and 50 captured in South Dakota, and 49 during 2018 and 74 during 2019. Average date of capture was 12 August (15 July–29 August); average capture date was 6 days earlier in 2019 (August 9) than 2018 (August 15). After aligning 13,393 movements made from 20 September–28 February 2018 and 2019 into three distance categories using k‐means cluster analysis, we used breakpoints between clusters to identify local (<25 km), regional (25–310 km), and migration (>310 km) movements. Our final dataset included 147 migration and 415 regional movements from 97 birds (30 in 2018 and 67 in 2019) after removing regional movements occurring before each bird's initial migration within North and South Dakota (i.e., postbreeding movements) and data from 26 birds wherein birds were shot by hunters (3), died (3), or transmitters stopped providing data (20) before leaving the Dakotas.

Of the 97 birds, 95 had an initial migration event (two birds moved south at distances <310 km) and 38 birds made later movements qualifying as subsequent migration movements, averaging 1.4 per bird (Range: 1–4, SD = 0.7). In 2018, 27 birds totaled 147 regional moves (average = 5.4, median = 5, SD = 4.1, Range: 1–15), and 57 birds totaled 268 regional moves (average = 4.7, min = 1, max = 13, median = 4, SD = 3.4) in 2019. Residency time between migration or regional moves averaged 10.2 days overall (SD = 13.2), was similar between years (10.2 and 10.3 days during autumn‐winters 2018–2019 and 2019–2020, respectively), was skewed right (median = 5), and varied substantially (Range: 1–78).

### 3.1 Movement timing and probability

Migration events occurred between 3 October and 24 February and averaged 23 November (median = 11 November). Timing of initial migration events occurred between 3 October and 6 December, with an average and median of 10 November during each year. Average and median initial migrations also were 10 November for males and females.

**FIGURE 2:**
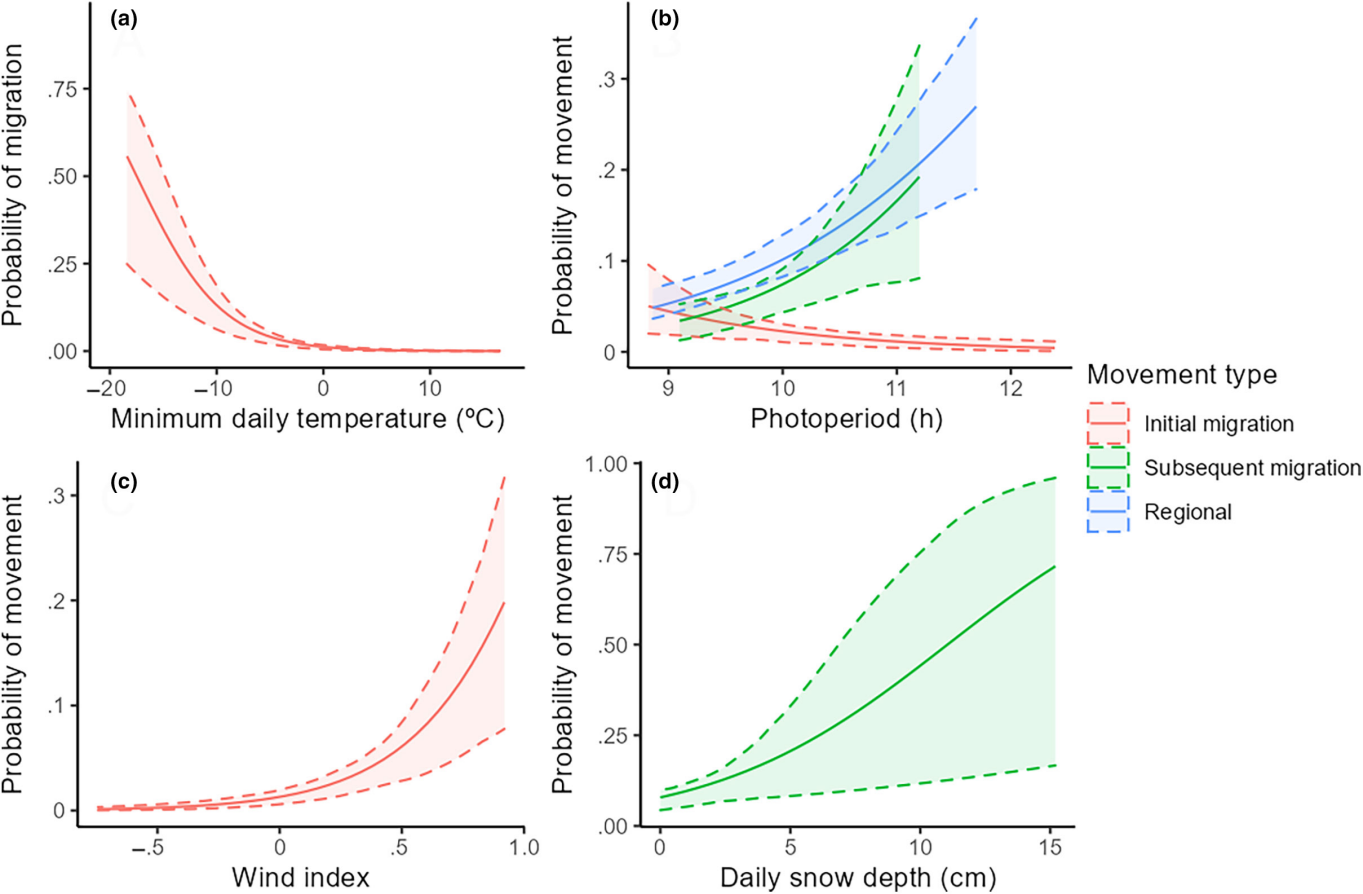
Predicted probability (95% confidence intervals) of juvenile Mallards initiating initial migration movements in relation to minimum daily temperature (a), initial migration, subsequent migration, and regional movements in relation to photoperiod (b), initial migration movements in relation to wind index (c), and initial migration movements in relation to daily snow depth (d) across the central United States during autumn‐winters 2018–2019 and 2019–2020.

The best‐competing model describing variation in migration movement timing included all predictor variables interacting with time (i.e., initial vs. subsequent migration movements), with the second‐best model being a model with all predictor variables interacting with latitude (ΔAIC = 5.3; Table 1). Minimum daily temperature was the most influential predictor of timing of initial migration movements (Table 2). Probability of making an initial migration event was low during days with minimum ambient temperatures >−5°C and increased 29% (95% CI: 20%–38%) for each 1°C reduction in daily minimum temperature (Figure 2a). Wind index was the second‐most important predictor variable influencing timing of initial migration events (Table 2). Wind bearings closer to migration direction (i.e., following or tailwinds) increased the probability of initial migration events (Figure 2c). Photoperiod was the third‐most important predictor variable influencing timing of initial migrations (Table 2). Probability of initiating an initial migration event was 2.0 (95% CI: 1.2–3.4) times more likely for each 1‐h decrease in photoperiod from the autumnal equinox to the winter solstice (Figure 2b). Probability of subsequent migration movements increased 80% (95% CI: 69%–93%) for every 1‐cm increase in daily snow depth (most influential predictor variable; Table 2; Figure 2d). Photoperiod was the second‐most influential predictor of subsequent migration events; probability of subsequent migrations was 40% (95% CI: 20%–80%) more likely for each 1‐h increase in photoperiod (Figure 2b). Subsequent migration events were 2.0 (95% CI: 1.1–3.6) times more likely during intervals including weekend days (Saturday and Sunday) compared to weekdays (Monday–Friday).

**TABLE 1:** Model selection results for logistic‐exposure model of daily probability of a juvenile Mallard remaining at a location during autumn‐winters 2018–2019 and 2019–2020.

Movement^a^	Model structure	*K* ^b^	ΔAIC^c^
Migration	Migration timing × (Sex + Minimum daily temperature + Cloud cover + Photoperiod + Daily snow depth + Wind index + Precipitation + Weekend)	19	0.0
	Latitude × (Sex + Minimum daily temperature + Cloud cover + Photoperiod + Daily snow depth + Wind index + Precipitation + Weekend)	19	5.3
	Sex + Minimum daily temperature + Cloud cover + Photoperiod + Daily snow depth + Wind index + Precipitation + Weekend	10	43.7
	Sex + Minimum daily temperature	4	88.0
	Sex + Daily snow depth	4	164.9
	Sex + Photoperiod	4	173.5
	Sex + Wind index	4	197.5
	Sex + Cloud cover + Precipitation	5	210.3
	Sex + Weekend	4	226.8
	Sex	3	227.6
Regional	Sex + Photoperiod	4	0.0
	Direction × (Sex + Minimum daily temperature + Cloud cover + Photoperiod + Daily snow depth + Wind index + Precipitation + Weekend)	18	8.2
	Sex + Minimum daily temperature + Cloud cover + Photoperiod + Daily snow depth + Wind index + Precipitation + Weekend	10	9.0
	Time × (Sex + Minimum daily temperature + Cloud cover + Photoperiod + Daily snow depth + Wind index + Precipitation + Weekend)	18	14.9
	Sex	3	27.0
	Sex + Wind index	4	28.0
	Sex + Weekend	4	28.6
	Sex + Daily snow depth	4	28.6
	Sex + Daily minimum temperature	4	28.7
	Sex + Precipitation + Cloud cover	5	29.4

^a^ Migration movement >310 km, regional movements between 25 and 310 km.

^b^ Number of estimated parameters.

^c^ Difference in Akaike's Information Criterion (AIC) value from model with the minimum AIC value; AIC of best migration model = 758.6; AIC of best regional model = 2171.3.

**TABLE 2:** Parameter estimates (*β*), 95% confidence intervals (CI), and relative likelihood (RL) when removing variables from models estimating daily probability of juvenile Mallards remaining at a location during autumn‐winters 2018–2019 and 2019–2020.

Predictor variable^a^	Initial migration move^b^				Subsequent migration moves^c^			
	*β*	Lower CI	Upper CI	RL^d^	*β*	Lower CI	Upper CI	RL
Intercept	4.27	3.65	4.89		2.81	2.17	3.45	
Sex (M)	0.21	−0.60	1.03	0.5	−0.45	−1.09	0.19	0.9
Temperature	1.40	1.01	1.79	3.1 × 10^11^	0.20	−0.16	0.55	0.6
Cloud cover	0.09	−0.23	0.42	0.4	−0.14	−0.47	0.18	0.5
Photoperiod	0.57	0.16	0.98	15.2	−0.74	−1.30	−0.18	8.4
Snow depth	−0.20	−0.43	0.03	1.7	−0.46	−0.76	−0.16	19.0
Wind index	−1.02	−1.38	−0.66	8.2 × 10^7^	−0.24	−0.55	0.08	1.1
Precipitation	0.18	−0.31	0.66	0.5	0.48	−0.10	1.06	2.8
Weekend	−0.36	−0.95	0.23	0.8	−0.67	−1.29	−0.05	3.0

^a^ Sex = Male (M) or Female (F); Temperature = minimum daily temperature (°C); Cloud cover = daily mean percentage cloud cover from 3‐h maximum of high‐ (>20,000 ft above ground level [AGL]), mid‐ (6500–20,000 ft AGL), and low‐level (<6500 ft AGL) cloud layers; Photoperiod = date‐ and location‐specific hours of daylight; Snow depth = daily maximum depth of snow cover (cm); Wind index = index of wind‐aided movement, where −1 represents a daily wind vector at the maximum of daily values and opposite of the movement bearing and 1.0 represents a daily wind vector at the maximum of daily values equal to the movement bearing; Precipitation = daily total precipitation accumulated at surface (cm); Weekend = proportion of days within the interval designated as a weekend day (Saturday or Sunday). Continuous predictor variables were scaled by subtracting the mean and dividing by the standard deviation.

^b^ Initial movement made >310 km.

^c^ Additional movements >310 km.

^d^ Relative likelihood.

Marked birds made an average of 2.7 (1–10) regional movements after their initial migration between 4 October and 26 February (median = 5 December). Median regional movements occurred on 4 December during autumn‐winter 2018–2019 and on 12 December during autumn‐winter 2019–2020. Median movements occurred on 5 December for males and 6 December for females.

The best‐competing model explaining variation in timing of regional movements included photoperiod and sex. The second‐best model was a model with all covariates interacting with the direction of movement (ΔAIC = 8.2; Table 1). Regional movements were 50% (95% CI: 39%–64%) more likely to occur with each 1‐h increase in photoperiod (Figure 2b).

### 3.2 Movement distance

Migration movements averaged 754 km (*n* = 147, SD = 358, median = 684) and ranged from 311 to 1779 km (2018–2019 average = 634, 2019–2020 average = 805). Initial migration movements averaged 861 km (*n* = 95, SD = 361, median = 838, 2018–2019 average = 747, 2019–2020 average = 909, female average = 981, male average = 758). Subsequent moves averaged 558 km (*n* = 52, SD = 258, median = 488, 2018–2019 average = 437, 2019–2020 average = 611, female average = 511, male average = 579). When we completed 10 models describing hypotheses explaining variation in migration movement distances, we found a model with minimum daily temperature better fit data than other models. The second‐best model was one that included all covariates (ΔAIC = 4.7; Table 3). Distance of migration events were longer during colder days (166 km SE = 26, 95% CI: 115–216 for each 1°C decrease in minimum daily temperature), and 147 km longer (SE = 56, 95% CI: 37–256) for females compared with males. Differences in daily temperatures of departure and arrival locations for initial migration movements averaged 5.0°C (SD = 5.0) minimum daily and 7.6°C (SD = 5.5) maximum daily temperatures. Distance of initial migrations was positively correlated with differences in minimum (*r* = .65) and maximum (*r* = .64) daily temperatures of departure and arrival locations.

**TABLE 3:** Model selection results for distance juvenile Mallards moved after initiating their initial migration, subsequent migrations, and regional movements during autumn‐winters 2018–2019 and 2019–2020.

Movement^a^	Model structure	*K* ^b^	ΔAIC^c^
Migration	Sex + Minimum daily temperature	5	0.0
	Sex + Minimum daily temperature + Cloud cover + Photoperiod + Daily snow depth + Wind index + Precipitation + Weekend	11	4.7
	Latitude × (Sex + Minimum daily temperature + Cloud cover + Photoperiod + Daily snow depth + Wind index + Precipitation + Weekend)	20	7.6
	Migration timing × (Sex + Minimum daily temperature + Cloud cover + Photoperiod + Daily snow depth + Wind index + Precipitation + Weekend)	20	13.0
	Sex + Wind index	5	23.8
	Sex + Cloud cover + Precipitation	6	26.8
	Sex + Photoperiod	5	31.4
	Sex + Daily snow depth	5	33.4
	Sex	4	33.6
	Sex + Weekend	5	35.0
Regional	Sex	4	0.0
	Sex + Minimum daily temperature	5	0.8
	Sex + Daily snow depth	5	1.4
	Sex + Photoperiod	5	1.8
	Sex + Wind index	5	2.0
	Sex + Weekend	5	2.1
	Sex + Cloud cover + Precipitation	6	3.9
	Sex + Minimum daily temperature + Cloud cover + Photoperiod + Daily snow depth + Wind index + Precipitation + Weekend	11	13.1
	Direction × (Sex + Minimum daily temperature + Cloud cover + Photoperiod + Daily snow depth + Wind index + Precipitation + Weekend)	19	24.9
	Time × (Sex + Minimum daily temperature + Cloud cover + Photoperiod + Daily snow depth + Wind index + Precipitation + Weekend)	19	25.0

^a^ Migration movement >310 km, regional movements between 25 and 310 km.

^b^ Number of estimated parameters.

^c^ Difference in Akaike's Information Criterion (AIC) value from model with the minimum AIC value; AIC of best migration model = 2110.1; AIC of best regional model = 2169.2.

Regional movement distances averaged 80 km (*n* = 194, SD = 64, median = 56) and ranged from 25 to 306 km. Average regional movement distances were 75 km in autumn‐winter 2018–2019, 84 km in autumn‐winter 2019–2020, 77 km for females, and 82 km for males. The null model, which included only the effect of sex was the best model fitted in this analysis, indicating that no predictor variable explained substantial variation in distance traveled (Table 3).

### 3.3 Movement direction

**FIGURE 3:**
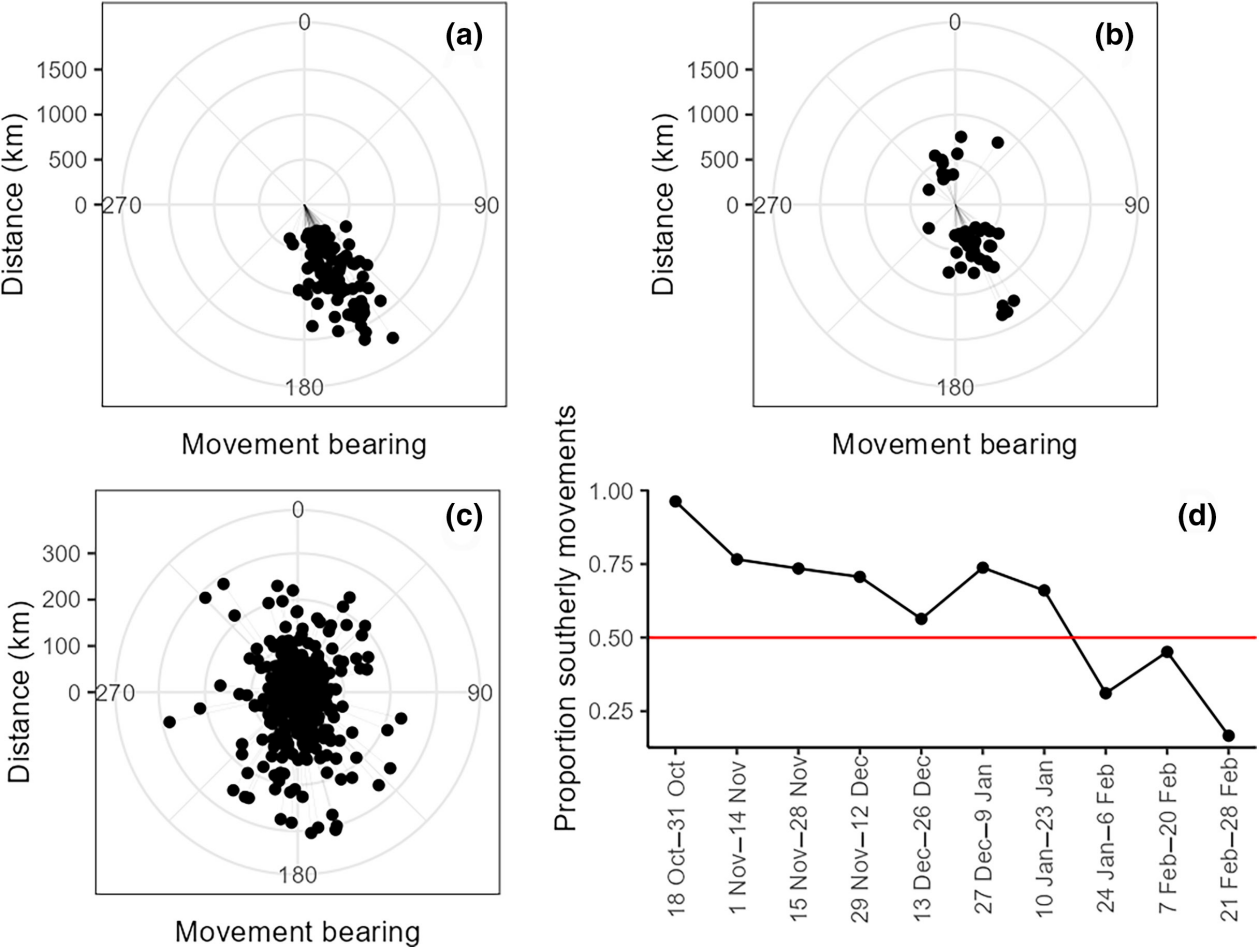
Direction of movements made by juvenile Mallards for initial migrations (a), subsequent migrations (b), and regional movements (c) across the central United States during autumn‐winters 2018–2019 and 2019–2020 (0° = north, 90° = east, 180° = south, and 270° = west). Proportion of migration and regional movements in a southerly direction (bearings 90–270°) between 18 October and 28 February during autumn‐winters 2018–2019 and 2019–2020 (d).

Migration movements followed predominately a southeastern direction (88%), with the remaining movements aligning to southwestern (4%) and northern (8%) directions (Figure 3a,b). Initial migration events were predominately southeastern (97%; Figure 3a). The 11 subsequent migration movements in a northern direction occurred in December (one event), January (four events), and February (six events).

Bearings of regional movements were distributed relatively equally among our primary directions (northeast = 21%, northwest = 23%, southeast = 31%, southwest = 25%; Figure 3c). Average regional movement distances were also similar with respect to direction (northeast = 75 km, northwest = 74 km, southeast = 83 km, southwest = 90 km).

Migration and regional movements to the south (between 90° and 180°) were more common (66%) than northerly movements during autumn‐winter. The proportion of southerly movements decreased steadily after mid‐October, with an abrupt switch from predominately southerly to northerly movements after 18–23 January (Figure 3d).

### 3.4 Annual distribution

**FIGURE 4:**
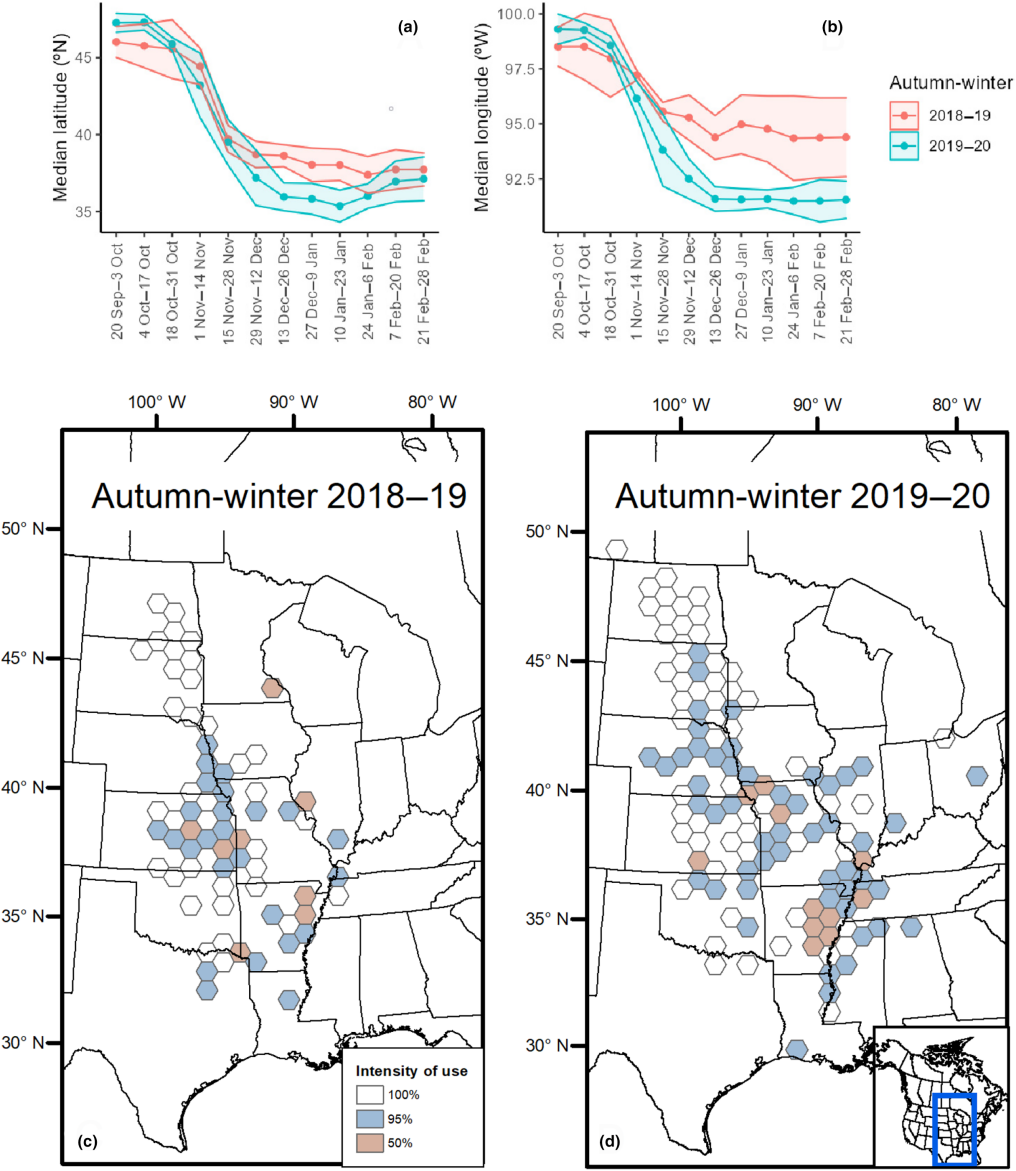
Median latitude (a) and longitude (b) of juvenile Mallards marked in North and South Dakota, USA, by fortnight during autumn‐winters 2018–2019 and 2019–2020. Cumulative percentage of days marked Mallards were present in locations (intensity of use) across the central United States during autumn‐winters 2018–2019 (c) and 2019–2020 (d).

Median latitude (°N) and longitude (°W) decreased between late October (18–31 October) and late November (15–28 November) during autumn‐winters 2018–2019 and 2019–2020 (Figure 4a,b). Median latitude of locations diverged between years, beginning mid‐December (13–26 December), with median Mallard locations 2.2–2.7° (~250–300 km) farther north during autumn‐winter 2018–2019 compared to 2019–2020. However, median latitude increased in autumn‐winter 2019–2020 later in the season, in late January, and the annual difference decreased to 0.6–1.4° (~65–150 km). As with latitude, median longitude of locations diverged between years beginning late November (29 November–12 December) to a difference of 2.8° (~245 km) farther west during autumn‐winter 2018–2019 compared to 2019–2020. This longitudinal difference persisted throughout the remainder of the seasons, with yearly differences reaching a maximum of 3.4° (~300 km) in late December (27 December–9 January). Intensity of use across autumn‐winters also reflected a pattern of greater use of birds north and west during autumn‐winter 2018–2019 compared to 2019–2020. For example, eastern Kansas was more intensely used in autumn‐winter 2018–2019, whereas portions of Arkansas and the Mississippi Alluvial Valley were more intensely used during autumn‐winter 2019–2020 (Figure 4c,d).

### 3.5 Harvest rate and distribution of harvest

Ten of 57 juvenile Mallards marked in 2018 (30 female, 27 male), were recovered and reported by hunters during the 2018–2019 hunting season, resulting in overall harvest rates of 10% for females and 26% for males. Three (one female, two male) of these 10 were harvested in North or South Dakota, resulting in a distribution of harvest in capture states of 33% for females and 29% for males. Hunters harvested seven birds (two female, five male) in Arkansas (2), Kansas (2), Iowa (1), Missouri (1), and Tennessee (1), resulting in distribution of harvest of 67% for females and 71% for males in migration and wintering states during the 2018–2019 waterfowl hunting season.

Twenty‐two of the 80 juvenile Mallards marked during 2019 (35 female, 45 male) were harvested and reported during the 2019–2020 waterfowl hunting season, resulting in overall harvest rates of 23% for females and 31% for males. Hunters harvested two females in North or South Dakota, resulting in a female distribution of harvest of 25% in capture states. Distribution of harvest in migration and wintering states was 75% for females and 100% for males in Arkansas (9), Missouri (3), Iowa (3), Mississippi (2), Illinois (1), Kansas (1), and Louisiana (1) during the 2019–2020 waterfowl hunting season.

## 4 DISCUSSION

### 4.1 Movement timing, distance, and direction

We found that Mallards did not leave their natal areas and vacate mid‐latitude locations exclusively because of cold and snowy conditions or primarily in association with weather favorable for flight, which is contrary to findings in recent studies (O'Neal et al., 2018; Weller et al., 2022). Migration and regional movement decisions by Mallards during autumn‐winter were related to factors supporting ambient conditions related to thermoregulation, favorable flight conditions, and factors unrelated to weather conditions. Therefore, although single hypotheses offer simpler explanations, we found multiple hypotheses provided greater explanatory power to describe varied large‐scale movement decisions of juvenile midcontinent Mallards. We believe our differing results may be partially related to our decision to split movements >25 km into multiple categories (i.e., initial migration, subsequent migration, and regional movements), because factors influencing autumn‐winter movements varied in magnitude and direction depending on movement type. Environmental conditions, specifically minimum daily temperature and favorable winds had the greatest association with timing of initial migration events, whereas these factors had less influence on timing of other long‐distance movements. Although inclement weather was a primary motivating factor for birds initiating initial migration movements out of the Dakotas, such conditions may have lacked the required intensity to motivate movements as birds progressed southward to lower latitudes where they initiated subsequent migration and regional movements. Subsequent migration movement timing included the greatest diversity of influential factors, indicating these movements were induced by a combination of factors intermediate to other movement types.

Mallard movement decisions of juvenile birds did not conform exclusively to the concept of facultative migrants, as photoperiod was associated with departure decisions of each movement type. Endogenous control of autumn migration movements via photoperiod has been established in some species of passerines (Newton, 2007), whereas our result was relatively novel for waterfowl except for Blue‐winged Teal (*Spatula discors*; Van Den Elsen, 2016). Our finding of photoperiod influencing long‐distance movements supports the premise that movements and ultimately distribution are under both endogenous and exogenous control, with effects varying from weak for initial migrations largely originating from the Dakotas, moderate for subsequent migrations, and most influential for regional movement, the latter two movements originating more frequently across mid and southern latitudes of the study area (Figure 1). This varied influence of photoperiod during autumn migration and into winter indicates that as birds move south, weather events had a waning influence on movement decisions and distribution, potentially because such weather conditions were mild enough to allow Mallards to maintain acceptable energy balance. The varying direction of photoperiod effects highlighted that Mallards were most sedentary during times of minimal daylength surrounding the winter solstice. Supporting this notion, average locations were relatively constant within weeks previous to and after the winter solstice (mid‐December–late January; Figure 4a,b). Therefore, the likelihood of Mallards making long‐distance movements into southern wintering areas became increasingly unlikely within weeks after the winter solstice. By early January, weather events that formerly would have influenced migration during November and December required substantially greater intensity to increase the probability of long‐distance movements into wintering areas, due to the shift in photoperiod to increasing daylength.

Snow depth, a factor identified as influencing food availability (Jorde et al., 1984), had a varied effect on timing of movement events. Weller et al. (2022) found snow cover duration and depth to influence timing of migration events for midcontinent Mallards during 2004–2011, whereas we found subsequent but not initial migration events were influenced by snow cover. Differing results may have been because we included movements >25 km occurring in all directions rather than limited to only southerly directions, and our inclusion of movements over a longer timeframe within autumn‐winter (end of February compared with end of December). Nonetheless, timing of initial migration events, which had the greatest correspondence with the sample used by Weller et al. (2022), had the least association with snow depth of the movement types we identified. We suspect that early snowfall in natal areas before sustained below‐freezing ambient temperatures may not singularly motivate migration decisions because snow cover generally does not persist due to warmer ground temperatures. Moreover, wetlands remaining unfrozen and available during these initial snow events would allow birds access to wetland‐based food sources. Motivation to minimize energy expenditure may influence timing of long‐distance movements to a greater extent earlier in autumn‐winter when birds were located at higher latitudes, whereas factors more associated with energy acquisition may be more motivating later in autumn‐winter when birds typically were located at lower latitudes. This interpretation supports an assumption of energetic carrying capacity models used for conservation planning for nonbreeding ducks, namely that food resources are the primary limiting resource (Reinecke & Loesch, 1996) and food availability influences wetland use by wintering ducks (Hagy et al., 2014).

Although environmental factors were included in the decision‐making process for movement timing, a potential proxy for human disturbance also was associated with decision‐making. Disturbance from humans or other causes can increase stress hormones (Casas et al., 2016), decrease feeding rates (Béchet et al., 2004), and affect daily behavioral patterns (Casazza et al., 2012; Cox Jr. & Afton, 1997). These behavioral and physiological responses can, in turn, decrease the overall body condition of individuals (Pearse et al., 2012; Szymanski et al., 2013; Zimmer et al., 2010). Ducks respond to disturbance by modifying distribution and habitat selection patterns (Fox & Madsen, 1997, Madsen & Fox, 1995, Cox Jr. & Afton, 2000, Stafford et al., 2007). Our finding that a potential index of human disturbance affected subsequent migration movements provides potentially novel insight into anthropogenic influences on avian behaviors and distributions (Cox Jr. & Afton, 2000; Jensen et al., 2016). Related, experimental disturbance events during autumn and winter reduced return rates of radio‐marked Mallards (Dooley et al., 2010), suggesting a higher percentage of birds permanently emigrated from the area when exposed to human disturbance. Although we find corroborating evidence that variation in disturbance is reflected in day of the week and our results matched predictions of a disturbance‐induced hypothesis, we were unable to verify that greater rates of disturbance occurred on weekends compared to weekdays across the range of marked birds. Therefore, we consider these results as providing preliminary support for this hypothesis that could be given more credence with additional investigations with more direct measures of disturbance.

Explanatory variables associated with timing of movements were generally independent of distance traveled. The primary exception was colder minimum daily temperatures being associated with greater distances of migration movements. Days with colder conditions, when thermoregulation was more energetically demanding, seemingly motivated birds to initiate and move greater distances to more favorable environmental conditions. Distance moved correlated positively with differences in daily temperatures at departure and arrival locations; thus, birds seemingly traded off extra energy expenditure required for migration flight with reduced thermoregulatory costs at arrival locations. Projected changes in winter distributions and total migration distances have been associated with increasing temperature (Masto et al., 2022; Notaro et al., 2016; Nuijten et al., 2020; Visser et al., 2009). Our results support these findings, providing a potential mechanism for how warmer conditions during times when Mallards make their initial migration related to shorter migration movements.

### 4.2 Consequences of movements

Annual variation in nonbreeding distributions is a common characteristic of facultative migrants using environmental conditions to drive movement decisions (Newton, 2007). We observed two cohorts of juvenile Mallards from the same natal region in the eastern Dakotas distribute themselves differently during consecutive wintering periods. Migration distances averaged ~170 km shorter during autumn‐winter 2018–2019, when average bird locations were ~250 km farther north and ~300 km farther west, compared to autumn‐winter 2019–2020. Varying winter distributions of Mallards in relation to winter temperatures have been inferred from band recoveries (Bellrose & Crompton, 1970; Lensink, 1964; Nichols et al., 1983), yet past studies cautioned that band recovery locations were imperfect representations of bird distributions because locations were dependent on hunter recoveries, which vary depending on spatial and temporal variation in hunting activity. Additionally, several weather‐related variables, including decreasing temperatures and snow cover, best explained many migration behaviors we modeled, including timing and distance of initial migration movements. Thus, our results broadly support the general conclusions from these earlier studies and expand our understanding of interannual distributional variation by identifying potential mechanisms.

Changes in autumn‐winter distribution of North American and European Mallards have been observed for decades (Meehan et al., 2021; Sauter et al., 2010). The more northwesterly distribution we observed during autumn‐winter 2018–2019 may become more common and the southeasterly distribution observed during autumn‐winter 2019–2020 may become less common in the future, as temperatures continue to increase and snowfall events, especially at mid‐latitude sites, become less common or less intense because of climate change (Guillemain et al., 2013; Notaro et al., 2014). Indeed, temperature and snow‐related variables were most influential on timing and distance of migration events, perhaps indicating later onsets of migration with initial staging distributions farther north and west as climate change progresses. Although midcontinent Mallards are likely to continue to use southern wintering regions such as the Mississippi Alluvial Valley during autumn‐winter, delayed and warmer winter conditions may reduce the frequency of large abundances of Mallards using historic regions in the southern portions of their nonbreeding range. Milder but increasingly variable autumn‐winter conditions may cause distributions to be less predictable annually, with large concentrations of Mallards found in varying locations across the midcontinent that possess wetlands and abundant forage.

Timing of migration and locations of nonbreeding destinations are strong selective pressures for migratory birds, and plasticity in migratory behaviors in response to short‐ and long‐term changes in external stimuli may result in resiliency of a species to climate change (Gilroy et al., 2016). Variation in timing or distribution, or lack thereof, may be potential risks to migratory species, especially if a phenological mismatch decouples the presence of individuals from seasonally abundant and critical resources (Møller et al., 2008). Alternatively, shortened migrations to areas that provide sufficient resources that meet energetic, physiological, and behavioral requirements could be beneficial. Gunnarsson et al. (2012) found that annual survival of Mallards in western Europe increased concurrent with reduced migration distance, speculating that survival increases may have resulted from decreased energetic demands of a shortened migration, although they could not rule out differences in harvest pressure that may have been more intense in southern compared to northern wintering areas. Indeed, we found harvest rate of midcontinent Mallards to be less in an autumn‐winter of shortened migration distances and a different autumn‐winter distribution. We suspect that the potentially different harvest rate resulted from a distribution coinciding with areas that may have reduced hunting activity, although we cannot rule out migration distance also may have indirectly influenced harvest rate if birds with reduced migration distance were in better body condition, making them less susceptible to harvest (Hepp et al., 1986). The Mississippi Alluvial Valley is a traditional and historic region for duck hunting, with abundant and high‐quality wintering waterfowl habitat (Reinecke et al., 1989) where more than a million waterfowl have been harvested each year (Raftovich et al., 2019). Therefore, an autumn‐winter distribution north and west of the Mississippi Alluvial Valley may have resulted in a decreased likelihood of harvest. Moreover, nonhunting mortality also has been hypothesized to be reduced when Mallards shorten migration and distribute north of traditional wintering sites (Aagaard et al., 2022).

Variable autumn‐winter distributions may have potential benefits for Mallard population dynamics and abundance, yet implications for harvest management and population dynamics of other species of ducks is less certain and may be deleterious. Status of the Mallard population is used to set duck harvest regulations for a suite of species that do not have individual harvest management plans. Although there are indications that many of these species also have modified their autumn‐winter distributions in response to a changing climate (Meehan et al., 2021; Van Den Elsen, 2016), the capacity of Mallards to modify their distributions compared to other duck species is likely greater because of their larger body size and propensity to exploit waste grain in upland agricultural fields (Baldassarre & Bolen, 1984), allowing Mallards to tolerate harsher environmental conditions than other species of ducks. Mallards are the most harvested duck species in the Central and Mississippi Flyways; therefore, if distributions of Mallards decouple from other duck species, then the waning buffering effect of Mallard harvest could create a situation where other duck species may be harvested at greater rates than in years when distributions are more aligned (Haugen et al., 2015). Therefore, climate change resulting in annually variable Mallard distributions may not only have consequences for hunter opportunities and satisfaction but also may affect harvest management of conspecifics.

Hunters desire waterfowl hunting seasons to occur concurrent with an abundance of animals present (Gruntorad et al., 2020). Regarding abundance, median latitude was unchanged (2018–2019) or increased (2019–2020) during late autumn‐winters (January–February). Mallards did not make more frequent long‐distance southward than northward movements during the end of January; thus, extending timing of duck hunting seasons beyond current regulations during years of this study would have provided negligible opportunities to hunt, even during winters when there are applicably greater numbers of Mallards in southern latitudes. Aerial surveys conducted in mid‐ and late winter in Mississippi and Arkansas also support this conclusion (Lehnen, 2013; Pearse et al., 2008). Moreover, continuation of hunting into February may result in harvest during initiation of nuptial pairing. Disruption of spring migration and potentially breaking mated pairs via harvest can result in reduced reproductive output for females (Lercel et al., 1999).

## 5 CONCLUSIONS

Our findings provide resource managers with information they may find useful when communicating with constituents regarding multiple factors that influence Mallard movements in autumn‐winter. Additionally, factors influencing movements themselves varied with respect to the type of long‐distance movement birds undertook. These factors in combination ultimately resulted in differing distributions of midcontinent Mallards during hunting seasons. Moreover, continued modification of behaviors based on changing environmental conditions to maximize their fitness may cause autumn‐winter distributions of Mallards to be more variable than experienced previously, with birds originating from North and South Dakota distributing themselves north and west of more traditional wintering regions in certain years. Resource managers attempt to time duck seasons to correspond with the greatest abundance of Mallards, but based on our two autumn‐winters of location data, shifting or extending hunting seasons would have had limited success at improving this correspondence at the most southerly regions in the United States. Modified and more variable autumn‐winter distributions may require conservation organizations from multiple regions to modify habitat‐management plans to account for greater variability in autumn‐winter ranges. For example, conservation planners may consider reevaluating distributions of conservation investments to match contemporary spatial distributions of Mallards more closely, or alternatively increase efforts in areas of historically high use to improve habitat quality and attractiveness. Finally, system change via climate change may create unexpected interactions between proposed regulations and variable distributions of Mallards that may result in observed harvest rates that disassociate with predicted values, and with the status and harvest rates of other species of ducks. Understanding these implications may be necessary for continued management of sustainable midcontinent populations of Mallards and other sympatric species of ducks.

## AUTHOR CONTRIBUTIONS

**Aaron T. Pearse:** Conceptualization (lead); formal analysis (lead); methodology (equal); project administration (equal); visualization (lead); writing – original draft (lead); writing – review and editing (equal). **Michael L. Szymanski:** Conceptualization (equal); funding acquisition (lead); methodology (equal); project administration (equal); writing – review and editing (equal). **Cynthia A. Anchor:** Conceptualization (equal); data curation (lead); methodology (equal); writing – review and editing (equal). **Michael J. Anteau:** Conceptualization (equal); project administration (equal); writing – review and editing (equal). **Rocco M. Murano:** Conceptualization (equal); funding acquisition (equal); writing – review and editing (equal). **David A. Brandt:** Conceptualization (equal); writing – review and editing (equal). **Joshua D. Stafford:** Conceptualization (equal); funding acquisition (equal); project administration (equal); supervision (equal); writing – review and editing (equal).

## FUNDING INFORMATION

This research was funded by North Dakota Game and Fish Department (funded in part by U.S. Fish and Wildlife Service Pittman‐Robertson Wildlife Restoration Act [CDFA# 15.611]) and the South Dakota Department of Game, Fish and Parks through the Federal Aid in Wildlife Restoration Fund, and South Dakota State University. C. Anchor also received financial support through a Waterfowl Research Foundation Fellowship from Ducks Unlimited, Canada's Institute for Wetland and Waterfowl Research.

## Supporting information


Data S1.

## Data Availability

Data are available as a U.S. Geological Survey data release in accordance with U.S. Federal regulations. https://doi.org/10.5066/P9GSUQUU.
