# Supplementary material for: Factors influencing autumn–winter movements of midcontinent Mallards and consequences for harvest and habitat management
Source: Ecol Evol. 2023 Oct 25;13(10):e10605. doi: 10.1002/ece3.10605 (PMC10600409; doi:10.1002/ece3.10605)

Supplemental information

Table S1. Univariate summaries of predictor variables used to model daily probability of a juvenile Mallard remaining at a location during autumn-winters 2018-19 and 2019-20.

|  | Migration movements^a^ | | | | |  | Regional movements^b^ | | | | |
| --- | --- | --- | --- | --- | --- | --- | --- | --- | --- | --- | --- |
| Predictor variable | Mean | SD | Median | 5%^c^ | 95%^d^ |  | Mean | SD | Median | 5% | 95% |
| Minimum daily temperature | -1.7 | 5.5 | -1.8 | -11.2 | 7.0 |  | -1.4 | 5.9 | -1.6 | -11.3 | 9.0 |
| Daily snow depth | 0.9 | 2.1 | 0.0 | 0.0 | 5.2 |  | 0.7 | 2.1 | 0.0 | 0.0 | 5.1 |
| Daily precipitation | 0.2 | 0.5 | 0.0 | 0.0 | 1.0 |  | 0.2 | 0.6 | 0.0 | 0.0 | 1.5 |
| Mean daily cloud cover | 50.0 | 28.9 | 52.0 | 5.0 | 96.0 |  | 42.1 | 30.1 | 39.0 | 2.0 | 94.0 |
| Wind index | 0.11 | 0.32 | 0.12 | -0.39 | 0.63 |  | 0.03 | 0.28 | 0.03 | -0.41 | 0.49 |
| Photoperiod | 10.3 | 0.8 | 10.1 | 9.3 | 11.9 |  | 9.9 | 0.4 | 9.9 | 9.3 | 10.6 |

^a^ Movement >310 km.

^b^ Movements between 25 and 310 km.

^c^ 5^th^ percentile.

^c^ 95^th^ percentile.

Table S2. Correlations of predictor variables used to model daily probability of a juvenile Mallard remaining at a location during autumn-winters 2018-19 and 2019-20.

|  | Minimum daily temperature | Daily snow depth | Daily precipitation | Mean daily cloud cover | Wind index | Photoperiod |
| --- | --- | --- | --- | --- | --- | --- |
| Minimum daily temperature | - | -0.471 | 0.343 | 0.200 | -0.008 | 0.366 |
| Daily snow depth | -0.471 | - | -0.012 | 0.068 | 0.034 | -0.200 |
| Daily precipitation | 0.343 | -0.012 | - | 0.386 | 0.025 | 0.116 |
| Mean daily cloud cover | 0.200 | 0.068 | 0.386 | - | 0.108 | 0.047 |
| Wind index | -0.008 | 0.034 | 0.025 | 0.108 | - | 0.062 |
| Photoperiod | 0.366 | -0.200 | 0.116 | 0.047 | 0.062 | - |

Figure S1. Example of movements made by a juvenile Mallard marked in 2019, depicting migration (A), regional (B), and local movements (C) during autumn-winter 2019-20.


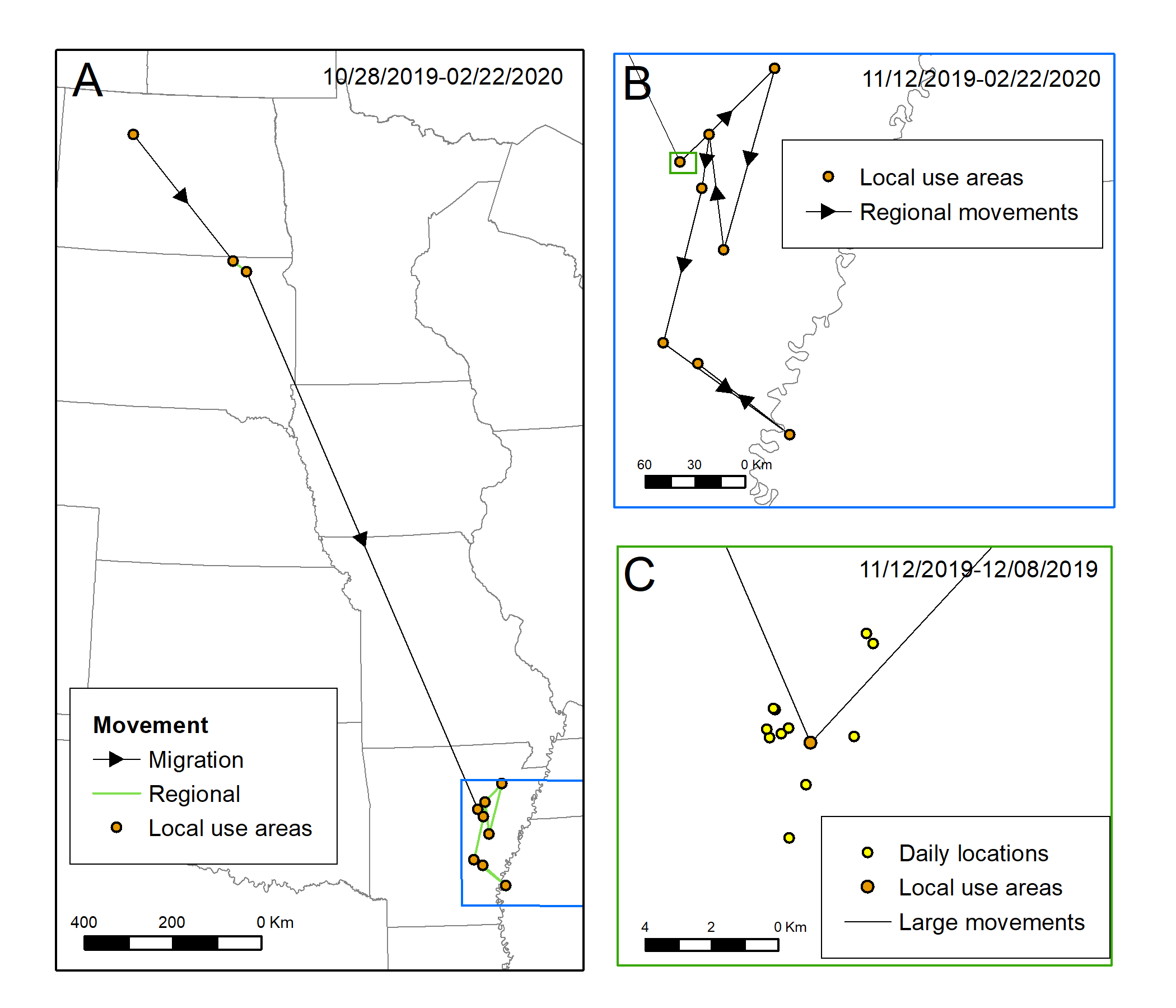

Supplement: Supplementary file 1 — Data S1. [file ECE3-13-e10605-s001.docx]
